# Requirement for the eIF4E Binding Proteins for the Synergistic Down-Regulation of Protein Synthesis by Hypertonic Conditions and mTOR Inhibition

**DOI:** 10.1371/journal.pone.0071138

**Published:** 2013-08-05

**Authors:** Michael J. Clemens, Androulla Elia, Simon J. Morley

**Affiliations:** 1 Department of Biochemistry and Molecular Biology, School of Life Sciences, University of Sussex, JMS Building, Falmer, Brighton United Kingdom; 2 Division of Biomedical Sciences, St George’s, University of London, Cranmer Terrace, London, United Kingdom; The John Curtin School of Medical Research, Australia

## Abstract

The protein kinase mammalian target of rapamycin (mTOR) regulates the phosphorylation and activity of several proteins that have the potential to control translation, including p70S6 kinase and the eIF4E binding proteins 4E-BP1 and 4E-BP2. In spite of this, in exponentially growing cells overall protein synthesis is often resistant to mTOR inhibitors. We report here that sensitivity of wild-type mouse embryonic fibroblasts (MEFs) to mTOR inhibitors can be greatly increased when the cells are subjected to the physiological stress imposed by hypertonic conditions. In contrast, protein synthesis in MEFs with a double knockout of 4E-BP1 and 4E-BP2 remains resistant to mTOR inhibitors under these conditions. Phosphorylation of p70S6 kinase and protein kinase B (Akt) is blocked by the mTOR inhibitor Ku0063794 equally well in both wild-type and 4E-BP knockout cells, under both normal and hypertonic conditions. The response of protein synthesis to hypertonic stress itself does not require the 4E-BPs. These data suggest that under certain stress conditions: (i) translation has a greater requirement for mTOR activity and (ii) there is an absolute requirement for the 4E-BPs for regulation by mTOR. Importantly, dephosphorylation of p70S6 kinase and Akt is not sufficient to affect protein synthesis acutely.

## Introduction

The polypeptide chain initiation factor eIF4E plays an important role in regulating the translation of capped mRNAs in eukaryotic cells and it is widely accepted that the availability of eIF4E to form the eIF4F initiation complex (comprising eIF4E, the large scaffold protein eIF4G and the RNA helicase eIF4A) can be a rate-limiting step in the initiation of protein synthesis [1]. The eIF4E-binding proteins 4E-BP1 and 4E-BP2 can bind eIF4E in competition with eIF4G and thus limit the formation of the eIF4F complex [2].

Initiation factor eIF4E is now considered to be a *bone fide* oncogene product [3], based on data from transgenic mouse studies [4] and the fact that many cancers have enhanced levels and/or activity of the protein [5]. High levels of eIF4E are able to confer resistance to apoptosis in cells exposed to a variety of death stimuli [6], [7], and eIF4E activity is regulated by the anti-apoptotic protein kinase Akt, an enzyme implicated in tumour cell survival and resistance to therapy [8]. Since the 4E-BPs inhibit the function of eIF4E by competing for the binding of eIF4G these small proteins often have opposite effects to those of eIF4E. Thus the 4E-BPs can revert the transformed phenotype in cells over-expressing eIF4E [9]. Moreover, cell cycle progression is blocked by over-expression of 4E-BP1 [10], most likely due to changes in the expression of proteins that regulate passage through the cell cycle. Consistent with this, 4E-BP1 can prevent the progression of cells from the G1 phase into S phase of the cell cycle without affecting the increases in cell mass or protein content characteristic of passage of cells through G1 [11]. Experimental knockdown of 4E-BP1 relieves the inhibition of cell cycle progression induced by cellular stresses such as hypoxia [12]. It is likely that an important mechanism of action of 4E-BP1 as an anti-oncogenic factor involves the induction of apoptosis, providing a counter-balance to the cell survival-promoting effects of eIF4E. Paradoxically, however, cells with decreased 4E-BP1 expression are less able to survive physiological stresses such as exposure to hypoxia or ionizing radiation [13], and it is possible that the inhibition of translation caused by disruption of eIF4F complex formation during hypoxia [14] may have a protective effect. A role for the 4E-BPs as factors that protect cells (and thus favour cell survival) under conditions of physiological stress has been suggested in earlier studies [15]. Relevant to this is the fact that the expression of 4E-BP1 (both phosphorylated and unphosphorylated) is elevated in a variety of tumours showing malignant progression [16].

The ability of the 4E-BPs to bind eIF4E is regulated by their state of phosphorylation, which in turn is controlled by the protein kinase mammalian target of rapamycin (mTOR). The latter exists in two complexes, mTORC1 and mTORC2, and is important in the control of a wide variety of pathways in health and disease [17]. In addition to the 4E-BPs, substrates for the mTOR complexes include the protein kinases p70S6 kinase [18] and Akt/protein kinase B [19]. Although these regulatory mechanisms are well understood it has been somewhat puzzling that inhibition of mTOR activity, which leads to the dephosphorylation of the 4E-BPs and marked inhibition of eIF4F assembly, often has little or no effect on the rate of overall protein synthesis in mammalian cells [20]. A possible interpretation of these observations is that, under optimal conditions, there is little requirement for cap binding by the eIF4F complex for the continuing translation of the majority of mRNAs. This may be a consequence of the functional “circularization” of polysomes, in which the 3′ end of the mRNA becomes associated with the 5′ end, allowing direct reinitiation of translation without the need for eIF4E to interact again with the cap structure [21]. We have hypothesized that cellular stresses that inhibit translation and lead to the disruption of polysome circularization should therefore increase the dependence on eIF4E and thus sensitize protein synthesis to inhibition of mTOR. Using the allosteric mTOR inhibitor rapamycin, the active site inhibitors Ku-0063794 [22] and PP242 [23] and the dual PI3-kinase/mTOR inhibitor PI-103 under conditions of optimal growth or physiological stress (hypertonic conditions or serum deprivation), we now provide evidence that supports this hypothesis. Moreover, although mTORC1 and mTORC2 have many direct and indirect substrates and targets, several of which have the potential to regulate global rates of protein synthesis, our data show that the acute effects of mTOR inhibition on translation require the 4E-BPs and that the dephosphorylation of other key mTOR substrates is not sufficient to impair overall protein synthesis, at least in the short term. These findings are relevant to our understanding of the role of the 4E-BPs in regulating the malignant phenotype, as well as the therapeutic responses to mTOR inhibitors of tumour cells with deregulated PI3-kinase, Akt or mTOR activity.

## Methods

### Materials

Tissue culture materials were from Gibco Life Technologies Ltd. (Paisley, U.K.) and GE Healthcare (Little Chalfont, U.K.). The mTOR inhibitors Ku-0063794, rapamycin, PP242 and PI-103 were from Tocris Bioscience (Bristol U.K.). m^7^GTP-Sepharose beads were from GE Healthcare. [^35^S]methionine was supplied by MP Biomedicals (Cambridge, U.K.). Antibodies against total 4E-BP1 and phosphorylated 4E-BP1 (Ser^64^) were from Santa Cruz Biotechnology Inc. (Heidelberg, Germany) and Cell Signalling Technology (Hitchin, Herts, UK) respectively. Antibodies against total Akt and phosphorylated Akt (Ser^473^) and against total p70S6K and phosphorylated p70S6K (Thr^389^ and Thr^421^/Ser^424^) were from Cell Signalling Technology. The antibody against GAPDH was from Merck Millipore (Feltham, U.K.). Anti-eIF4GI was produced in-house. Horseradish peroxidise-linked secondary antibodies were from Cell Signalling Technology. PVDF membrane and rainbow markers were supplied by GE Healthcare.

### Cell Lines

Mouse embryonic fibroblasts (MEFs) with a double knockout of the 4E-BP1 and 4E-BP2 genes [24] and their corresponding wild-type controls were a gift from Dr Nahum Sonenberg (McGill University, Montreal, Canada). MEFs with a Ser to Ala mutation at position 51 of the eIF2α gene (S51A cells) [25] and their corresponding wild-type controls were a gift from Dr Randall Kaufman (Wayne State University, Ann Arbor, Michigan, USA).

### Cell Culture and Treatments

The cells were maintained in monolayer cultures at 37°C in humidified air with 5% CO_2_ in Dulbecco’s modified Eagle medium supplemented with penicillin (50 units/ml), streptomycin (50 units/ml) and 10% foetal bovine serum. Cultures were split every 3–5 days and all experiments were performed on cells that were in exponential growth. To induce hypertonic stress the cells were incubated for 1 h in the above medium containing additional NaCl (0.1 M except where otherwise indicated). For experiments involving serum deprivation the cells were washed with phosphate buffered saline (PBS) and then incubated in the above medium with or without serum for 24 h. The cells were then incubated without or with rapamycin (100 nM), Ku-0063794 (1 µM), PP242 (5 µM) or PI-103 (5 µM) for 1 h.

### Protein Synthesis

Following the treatments described above overall protein synthesis in intact cells was measured by the incorporation of [^35^S] methionine (1–2 µCi/ml for 1 h) into trichloroacetic acid-insoluble material as described previously [26]. Total cellular protein content was determined and rates of protein synthesis calculated as counts per min incorporated per µg protein.

### Immunoblotting

Cells were washed in PBS and subjected to lysis as previously described [27]. Nuclei were removed by centrifugation, equal amounts of cytoplasmic protein were fractionated by electrophoresis on sodium dodecyl sulphate (SDS) polyacrylamide gels and the proteins were then transferred to PVDF paper. The blots were blocked, incubated with primary antibodies, thoroughly washed and then incubated with horseradish peroxidase-linked secondary antibodies as described [27]. Binding of the latter was detected by enhanced chemiluminescence, using Lumiglo reagent (Cell Signalling Technology) according to the manufacturer’s instructions.

### m^7^GTP-Sepharose Affinity Purification

Initiation factor eIF4E and its associated proteins were isolated from cell extracts (containing equal concentrations of protein) by affinity chromatography on m^7^GTP-Sepharose beads as described [28]. The beads were washed thoroughly with ice-cold buffer (20 mM MOPS-KOH, pH 7.4, 75 mM NaCl, 1 mM Mg acetate, 7 mM 2-mercaptoethanol, 0.25% Igepal, 1 µM microcystin, 2 mM benzamidine, 0.1 mM GTP, 10 mM NaF) and the bound proteins eluted with SDS gel sample buffer. The proteins were then analyzed by immunoblotting as described above.

### Statistical Analysis of Data

The data from the protein synthesis determinations are shown as the means ± S.E.M. Independent experiments were performed at least three times and typical examples are presented. Unpaired t tests (Prism 3 software, GraphPad) were used to determine statistical significance and p values of <0.05 are considered to demonstrate significant differences.

## Results

**Figure 1:**
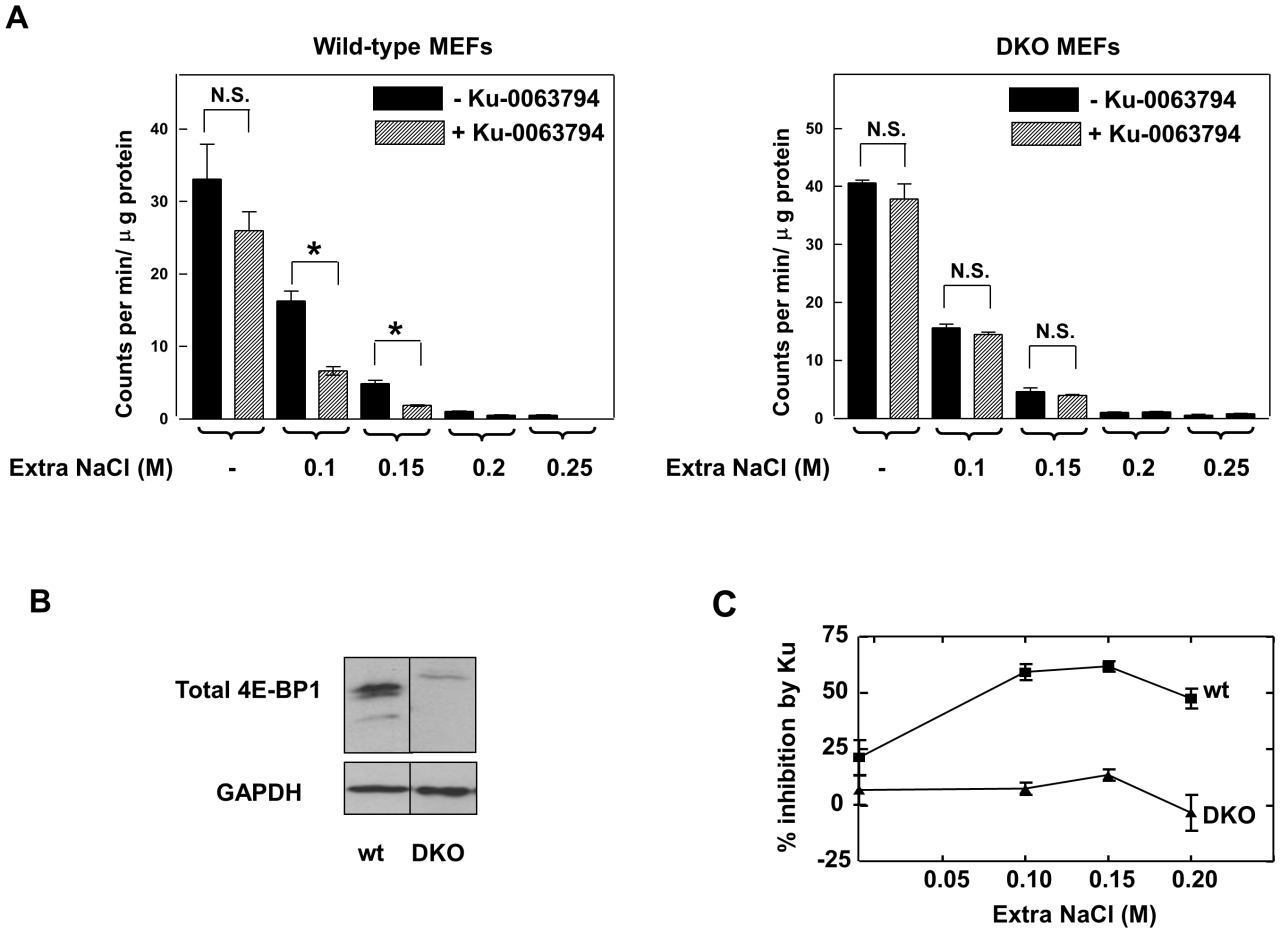
Influence of salt concentration on the inhibition of protein synthesis by Ku-0063794 in 4E-BP wild-type cells and cells with a double knockout of 4E-BP1 and 4E-BP2. (A) Wild-type and DKO MEFs were pre-incubated in complete medium in the presence of additional NaCl at the concentrations indicated. After 1 h the cells were further incubated with or without Ku-0063794 (1 µM) for 1 h and total protein synthesis was then measured by the incorporation of [^35^S]methionine (2 µCi/ml) for 1 h as described in Experimental. The data are the means of triplicate determinations and are expressed as counts per min incorporated per µg of protein ± S.E.M. Significances of differences between incubations ± Ku-0063794 were determined by unpaired t tests: * = p<0.005; N.S. = not significant. (B) Extracts from 4E-BP wild-type and DKO cells were analysed for expression of 4E-BP1 by immunoblotting. Blots for GAPDH are also shown as loading controls. (C) Summary of % inhibition of protein synthesis by Ku-0063794 in 4E-BP wild-type (wt) and DKO cells as a function of the additional NaCl concentration.

It is well established that the stress imposed on cells by mildly hypertonic conditions results in a marked inhibition of protein synthesis. However the consequences of inhibition of mTOR activity under such conditions have not previously been examined. Accordingly we investigated the effect of the mTOR inhibitor Ku-0063794 on translation in murine embryonic fibroblasts in the presence of increasing salt concentrations. Figure 1A (left panel) confirms the sensitivity of overall protein synthesis in wild-type MEFs to the hypertonic conditions imposed by additional NaCl in the culture medium. The data show that whereas Ku-0063794 had only a small effect under normal salt conditions, which was not statistically significant, in the presence of additional NaCl (0.1 M or greater) the effect of Ku-0063794 was substantially increased (50–60% inhibition – statistically significant, p<0.005). The effects on protein synthesis of the mTOR inhibitor in the presence and absence of hypertonic conditions were also analysed by analysis of the distributions of ribosomes between polysomes and sub-polysomal fractions on sucrose gradients. Whereas there was very little decrease in the % of ribosomes in polysomes in response to 0.1 M NaCl or 1 µM Ku-0063794 individually, there was a noticeably greater effect when the cells were exposed to both treatments together (data not shown).

To determine the requirement for the 4E-BPs for the effects of Ku-0063794 on protein synthesis under normal and hypertonic conditions, similar experiments were performed on 4E-BP1/2 double knockout (DKO) cells. The latter cells are genetically disrupted for both 4E-BP1 and 4E-BP2 and express neither of these proteins [24]. The absence of 4E-BP1 is confirmed by the immunoblot shown in Figure 1B. The data in Figure 1A (right panel) demonstrate that protein synthesis in the DKO cells was just as sensitive as in 4E-BP wild-type cells to inhibition by hypertonic conditions; however, in these cells there was no significant effect of Ku-0063794 under any of the salt conditions tested. The relative responses of the wild-type and DKO cells to Ku-0063794 at different salt concentrations, as determined by [^35^S]methionine incorporation, are summarized in Figure 1C.

Similar experiments were carried out using MEFs with a serine to alanine mutation at position 51 of the α subunit of initiation factor eIF2 (S51A cells). These cells are unable to undergo phosphorylation at this site in response to a variety of physiological stresses, rendering them deficient in the regulation of polypeptide chain initiation [25]. Protein synthesis in the S51A cells remained sensitive to inhibition by increasing concentrations of NaCl (data not shown), indicating that phosphorylation of eIF2α is not required for the effect of hypertonic conditions on translation. Moreover, hypertonic conditions still significantly enhanced the effect of Ku-0063794 on protein synthesis in the S51A cells, unlike the situation with the 4E-BP DKO cells (Table 1). Overall, these data indicate that hypertonic conditions inhibit protein synthesis by a mechanism that requires neither inhibition of eIF4E by 4E-BP1/2 nor phosphorylation of eIF2α by stress-sensitive kinases, whereas the effect of Ku-0063794 under hypertonic conditions does require the presence of 4E-BP1 and/or 4E-BP2 but is independent of eIF2α phosphorylation.

**Table 1:** Contrasting requirements for 4E-BP expression or eIF2α phosphorylation for inhibition of protein synthesis by Ku 0063794 under normal and hypertonic conditions.

Cell line	Conditions	% inhibition of protein synthesis by Ku-0063794
4E-BP wild-type	Normal	8.3±4.7
4E-BP wild-type	Hypertonic	56.3±2.9 (p<0.0001)*
DKO	Normal	3.2±3.4
DKO	Hypertonic	3.4±2.4 (not significant)*
S51 wild-type	Normal	23.6±12.1
S51 wild-type	Hypertonic	63.6±6.0 (p = 0.01)*
S51A	Normal	34.0±4.5
S51A	Hypertonic	47.8±4.6 (p = 0.05)*

The indicated cell lines were pre-incubated in complete medium in the absence or presence of additional 0.1 M NaCl. After 1 h the cells were further incubated with or without Ku-0063794 (1 M) for 1 h and total protein synthesis was then measured by the incorporation of [^35^S]methionine (2 µCi/ml) for 1 h as described in Experimental. The data are the means of 6–9 independent determinations. Asterisks show the significance of the differences between the effects of Ku-0063794 on protein synthesis under normal versus hypertonic conditions.

**Figure 2:**
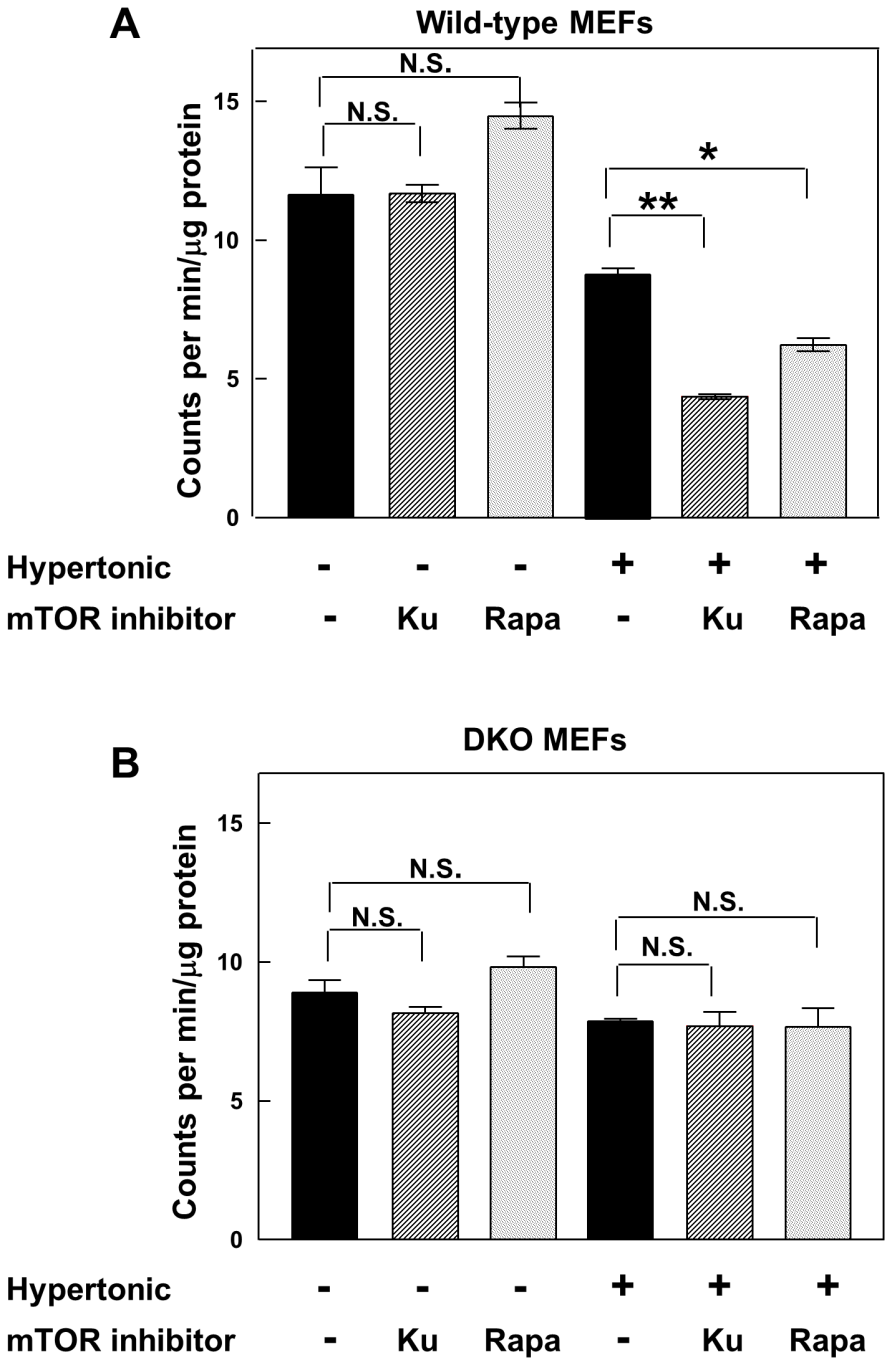
Comparison of the effects of Ku-0063794 and rapamycin on protein synthesis in 4E-BP wild-type and 4E-BP-deficient cells under normal and hypertonic conditions. (A) Wild-type cells and (B) DKO cells were pre-incubated in complete medium in the absence or presence of additional 0.1 M NaCl. After 1 h the cells were further incubated with or without Ku-0063794 (1 µM) or rapamycin (100 nM) for 1 h and total protein synthesis was then measured by the incorporation of [^35^S]methionine (2 µCi/ml) for 1 h as described in Experimental. The data are the means of triplicate determinations and are expressed as counts per min incorporated per µg of protein ± S.E.M. Significances of differences between incubations with and without each mTOR inhibitor were determined by unpaired t tests: * = p<0.002; ** = p<0.0001; N.S. = not significant.

We have extended these experiments to examine the effects of other mTOR inhibitors on overall protein synthesis in the absence or presence of additional salt. The well characterised mTORC1 inhibitor rapamycin failed to inhibit [^35^S]methionine incorporation at all under normal conditions but reduced protein synthesis by 29% in 4E-BP wild-type cells under hypertonic conditions (statistically significant, p<0.002) (Fig. 2A). As noted by others [29], [30], rapamycin was less effective than mTOR kinase inhibitors such as Ku-0063794, probably because there are rapamycin-resistant functions of mTORC1 [31]. In confirmation of the previous data, the DKO cells were completely resistant to inhibition of mTOR by either rapamycin or Ku-0063794 under both normal and hypertonic conditions (Fig. 2B). Similar results were obtained with the mTORC1/2 inhibitor PP242. In this case, the effect of the drug in 4E-BP wild-type cells was increased from 30% inhibition under normal conditions to 65% inhibition under hypertonic conditions (statistically significant). PP242 did not inhibit protein synthesis at all in DKO cells under either condition (our unpublished data).

**Figure 3:**
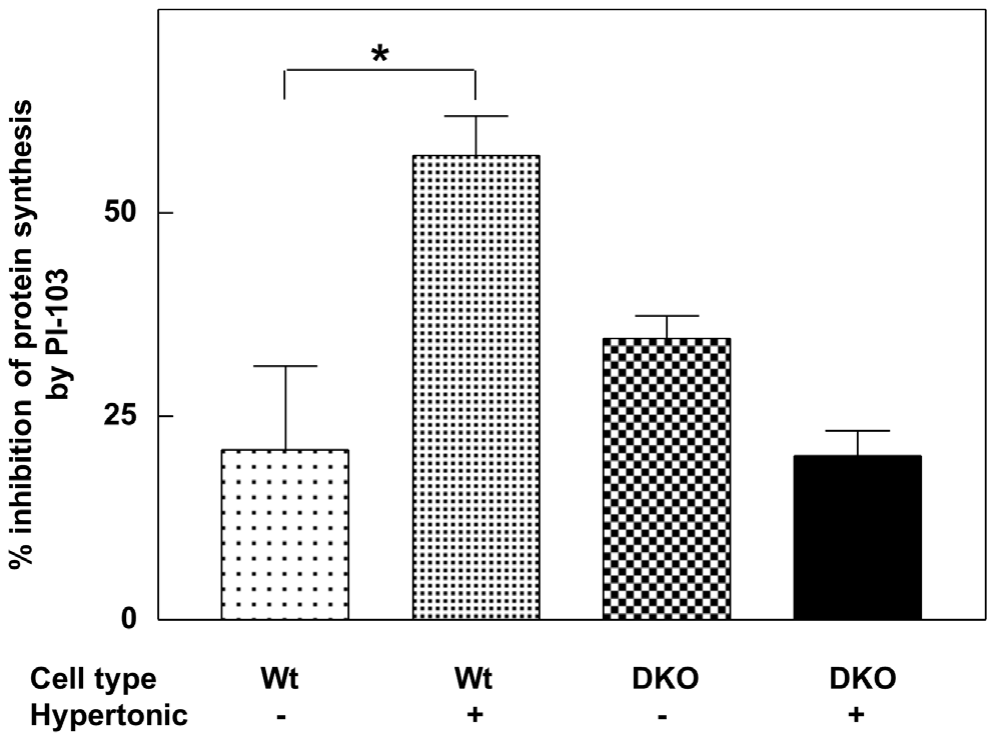
Influence of salt concentration on the inhibition of protein synthesis by PI-103. 4E-BP wild-type and DKO cells were incubated in complete medium in the absence or presence of additional 0.1 M NaCl. After 1 h the cells were further incubated with or without PI-103 (5 µM) for 1 h and total protein synthesis was then measured by the incorporation of [^35^S]methionine (2 µCi/ml) for 1 h as described in Experimental. The data are the means ± S.E.M of 9 independent determinations on 4E-BP wild-type cells and 6 independent determinations on DKO cells and are expressed as the % inhibition of protein synthesis by PI-103 in the absence or presence of the additional NaCl. Significance of difference was determined by unpaired t test: * = p<0.01.

Another compound that inhibits not only mTORC1/2 but also PI3-kinase is the pyridofuropyrimidine PI-103 [32]. We examined the sensitivity of overall protein synthesis to PI-103 in 4E-BP wild-type and DKO MEFs under both normal and hypertonic conditions (Fig. 3). Protein synthesis under optimal growth conditions was moderately inhibited by PI-103 in both cell types (20.9±10.2% and 34.6±2.8% inhibition respectively). However, as we observed with the other mTOR inhibitors, in the wild-type cells the presence of additional 0.1 M NaCl enhanced the inhibition by PI-103 (57.0±4.9%). This enhancement was statistically significant (p<0.01). Again there was no enhancement of inhibition by hypertonic conditions in the DKO cells (20.2±3.0% inhibition). These data indicate that, although PI-103 can partially inhibit protein synthesis in the absence of 4E-BP1 and 2 (presumably as a consequence of the inhibition of PI3-kinase activity), the 4E-BPs are needed for the salt-mediated enhancement of inhibition by PI-103.

**Figure 4:**
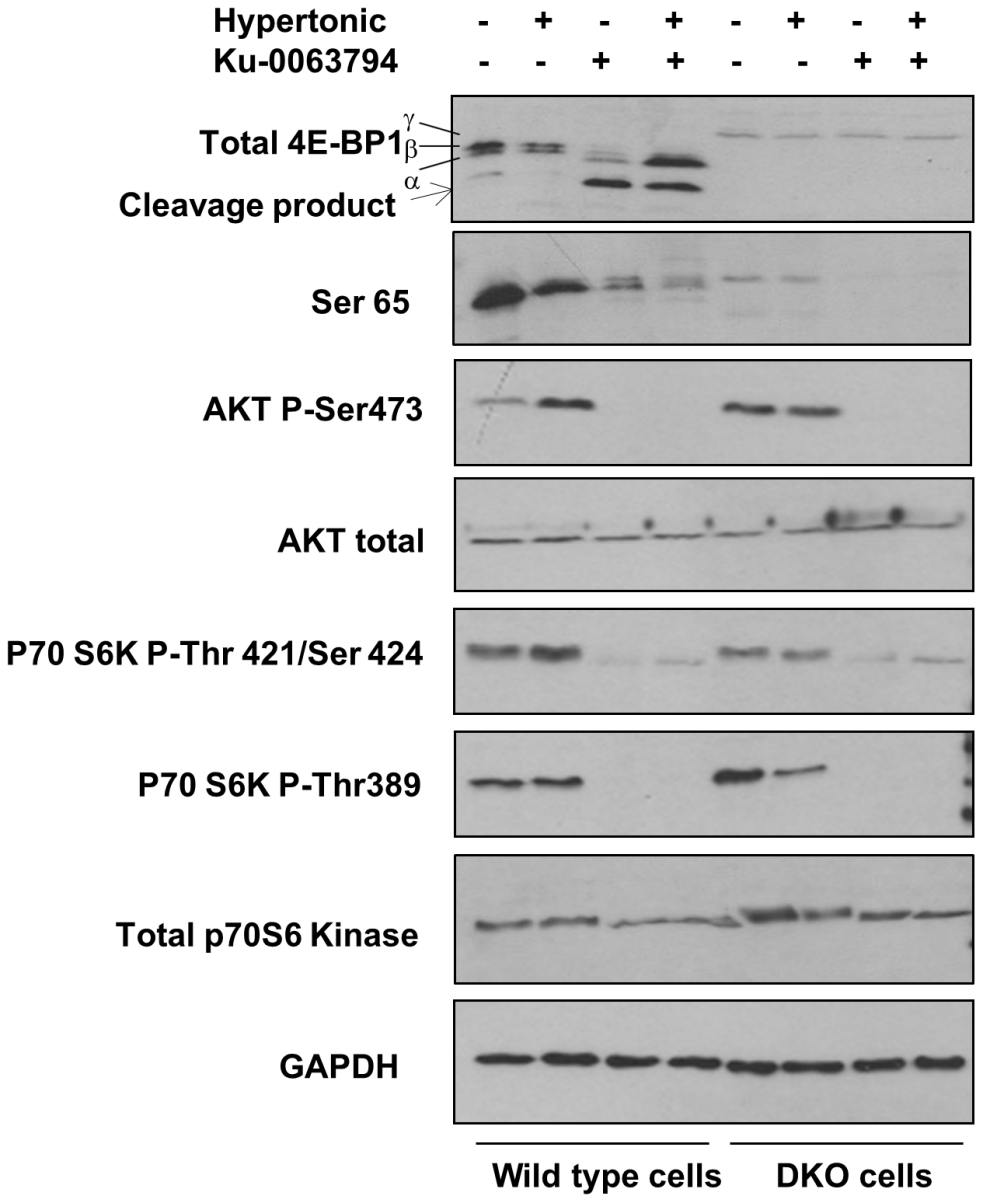
Inhibition of phosphorylation of 4E-BP1, p70S6 kinase and Akt by Ku-0063794 under normal and hypertonic conditions. 4E-BP wild-type and DKO MEFs were pre-incubated in complete medium in the absence or presence of additional 0.1 M NaCl. After 1 h the cells were further incubated with or without Ku-0063794 (1 µM) for 1 h and extracts were prepared. The extracts were analysed for total 4E-BP1 and phosphorylated 4E-BP1 (Ser^64^), total and phosphorylated Akt (Ser^473^), and total and phosphorylated p70S6kinase (Thr^421^/Ser^424^ and Thr^389^) by SDS gel electrophoresis and immunoblotting. The positions of the differentially phosphorylated α, β and γ forms and of a cleavage product of 4E-BP1 are indicated. Blots for GAPDH are also shown as loading controls.

**Figure 5:**
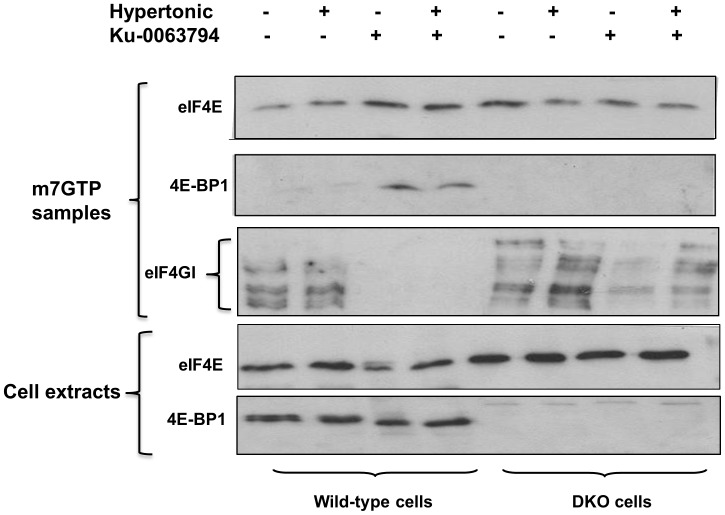
Inhibition of eIF4F complex formation by Ku-0063794 under normal and hypertonic conditions. 4E-BP wild-type and DKO MEFs were pre-incubated in complete medium in the absence or presence of additional 0.1 M NaCl. After 1 h the cells were further incubated with or without Ku-0063794 (1 µM) for 1 h and extracts were prepared. Using equal quantities of total protein, the extracts were then subjected to affinity chromatography on m^7^GTP-Sepharose to isolate eIF4E and its associated proteins, as described in Experimental. The bound proteins were analysed for eIF4E, 4E-BP1 and eIF4GI by SDS gel electrophoresis and immunoblotting.

Since 4E-BP1, when in a hypophosphorylated state, regulates cap-dependent translation by competing with eIF4G for binding to eIF4E, it was important to determine whether hypertonic conditions influence the state of phosphorylation of this protein. Figure 4 shows that, in the absence of Ku-0063794, 4E-BP1 in wild-type cells remained in a predominantly phosphorylated state under the higher salt conditions, as judged both by its mobility on SDS gels and by reactivity of the protein with an antibody against the Ser^64^ phosphorylation site. This is consistent with evidence that mTOR remains active under hypertonic conditions and indeed may be activated by osmotic stress [33], [34]. Incubation of the cells with Ku-0063794 caused extensive dephosphorylation of 4E-BP1, under both normal and hypertonic conditions. The immunoblots also revealed evidence of cleavage of 4E-BP1 in wild-type cells in the presence of Ku-0063794 but this was not enhanced by the higher salt conditions. Instead a stronger band corresponding to intact hypophosphorylated 4E-BP1 was seen. Consistent with the effect of Ku-0063794 on the state of phosphorylation of 4E-BP1, binding of the latter to eIF4E was strongly stimulated in the presence of the mTOR inhibitor (Fig. 5). Conversely, the association of eIF4GI with eIF4E in the eIF4F complex was completely eliminated by Ku-0063794, both under normal and hypertonic conditions. Additional NaCl alone had no effect on binding of eIF4GI to eIF4E (Fig. 5).

As expected, a 4E-BP1 signal was absent from the DKO cell extracts, although a faint band of slightly slower mobility was detected (Fig. 4). Since this protein cross-reacted with antibodies against both total 4E-BP1 and Ser^64^, and was dephosphorylated in the presence of Ku-0063794, it may correspond to 4E-BP3. There was no 4E-BP signal under any conditions when eIF4E and its associated proteins in DKO cells were analysed by immunoblotting (Fig. 5). In spite of this, some decrease in the association of eIF4GI with eIF4E occurred in these cells in the presence of Ku-0063794, although this was clearly not sufficient to impair overall protein synthesis (Figs. 1 and 2). Taken in combination with the protein synthesis results, these findings suggest that Ku-0063794-mediated dephosphorylation of 4E-BP1 and the consequent inhibition of eIF4F complex formation are not sufficient to inhibit overall translation in the short term. Moreover, the sensitization to Ku-0063794 in hypertonically stressed cells does not involve any greater extent of dephosphorylation of 4E-BP1 or inhibition of eIF4GI binding to eIF4E (the latter being completely eliminated by Ku-0063794 alone).

The lack of response of protein synthesis to mTOR inhibitors in the DKO cells indicates that 4E-BP1 and/or 4E-BP2 are necessary for the acute regulation of overall translation by the protein kinase. However, mTOR has many other targets with the potential for the control of protein synthesis and it was of interest to determine whether the regulation of these targets is disrupted in DKO cells. One such substrate is p70S6 kinase, which phosphorylates ribosomal protein S6 as well as several other proteins [35]. Immunoblotting analysis revealed that the phosphorylation of p70S6 kinase, both at Thr^421^/Ser^424^ and at Thr^389^– sites which regulate the activity of the enzyme [36] - was strongly inhibited by Ku-0063794 in 4E-BP wild-type and DKO cells, under both control and hypertonic conditions (Figure 4). Thus inhibition of the activity of p70S6 kinase is not sufficient to cause rapid down-regulation of overall protein synthesis. A similar conclusion can be drawn with respect to the mTORC2 substrate Akt (Figure 4). Thus, although phosphorylation of Akt at Ser^473^ regulates the ability of this enzyme to control long-term cellular responses such as proliferation and survival [37], these effects can be dissociated from the acute control of overall translation by mTOR in mouse fibroblasts.

**Figure 6:**
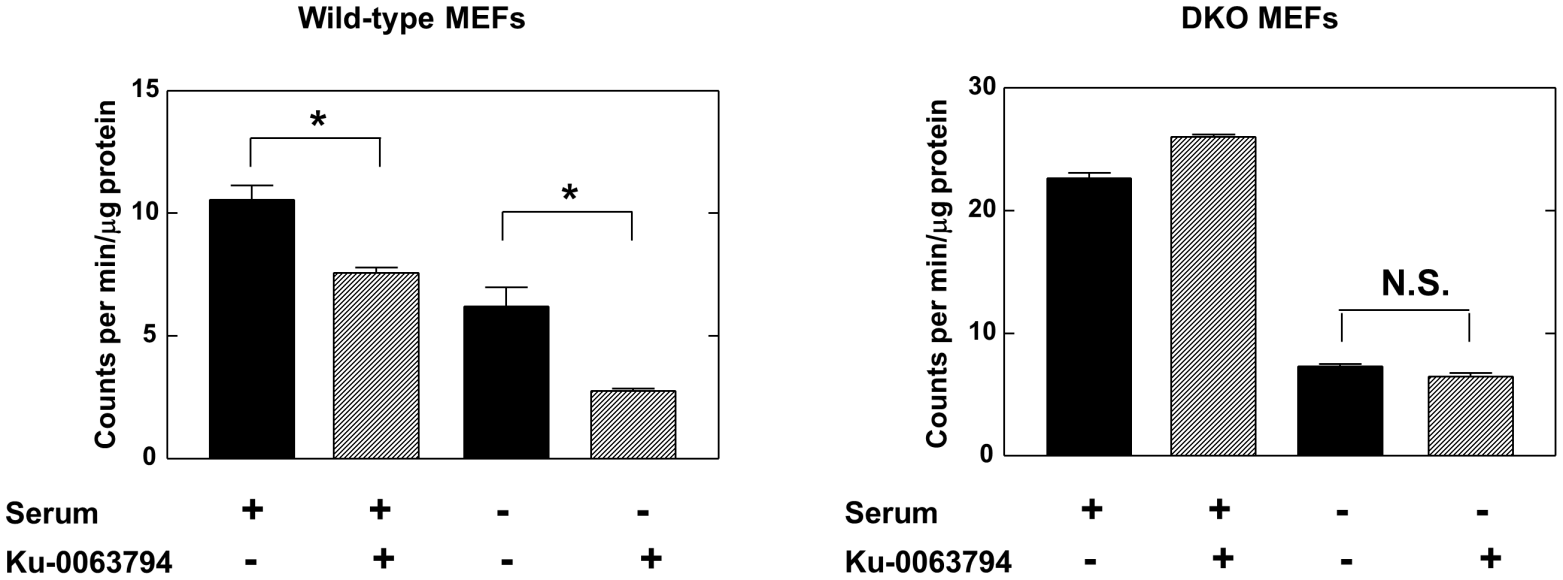
Effect of Ku-0063794 on protein synthesis in 4E-BP wild-type and 4E-BP deficient cells under normal and serum-deprived conditions. Wild-type and DKO cells were incubated for 24 h in complete medium or in medium lacking serum. The cells were incubated with or without Ku-0063794 (1 µM) for 1 h and overall protein synthesis was then measured by the incorporation of [^35^S]methionine (1 µCi/ml) for 1 h as described in Experimental. The data are the means of triplicate determinations and are expressed as counts per min incorporated per µg of protein ± S.E.M. Significances of differences between incubations ± Ku-0063794 were determined by unpaired t tests: * = p<0.05; N.S. = not significant.

We have investigated whether the sensitization of cells to the effects of mTOR inhibitors is specific to hypertonic conditions or whether other cell stresses may have similar effects. Figure 6 shows that serum starvation also sensitizes 4E-BP wild-type cells to inhibition of protein synthesis by Ku-0063794. In the experiment shown, although the wild-type cells had some sensitivity to the mTOR inhibitor under unstressed conditions (28% inhibition), the effect of Ku-0063794 was markedly increased after 24 h of serum deprivation (55% inhibition, significantly different from the value for the cells in the presence of serum, p = 0.0005). Again there was no significant inhibition by Ku-0063794 in the DKO cells, in the absence or presence of serum. These data suggest that at least some stresses other than hypertonicity, including the important one of growth factor deprivation, can also increase the sensitivity of overall protein synthesis to the regulation of the 4E-BPs by mTOR.

## Discussion

It has been known for many years that hypertonic conditions reversibly inhibit protein synthesis in mammalian cells [38] and a number of studies have addressed the molecular mechanisms and signalling pathways involved [39]–[41]. In spite of this work the basis for the inhibition of translation by additional salt remains unclear since neither of the principal mechanisms by which polypeptide chain initiation is regulated appear to be involved. High salt treatment does not cause any increase in the phosphorylation of the α subunit of initiation factor eIF2 [39] (and our unpublished data), nor any decrease in the formation of [40S ribosomal subunit.Met-tRNA_f_] initiation complexes [42], and cells with a non-phosphorylatable form of eIF2α (S51A MEFs) are no less susceptible than S51 wild-type cells to salt-mediated inhibition of protein synthesis (data not shown). This would suggest that a subsequent step in the initiation process, such as mRNA binding to ribosomes, is impaired. However our present work rules out a role for 4E-BP1 or 4E-BP2, and thus by implication the availability of the cap binding factor eIF4E, in the effect. This is consistent with the lack of any major effect of higher salt conditions on the state of phosphorylation of 4E-BP1 (Fig. 4), or on the association of the latter with eIF4E (Fig. 5), and with the ability of protein synthesis to recover from salt-mediated inhibition under conditions where the phosphorylation of 4E-BP1 is blocked [41]. Our results differ from those of Kwak et al. [34], who reported dephosphorylation of both 4E-BP1 and p70S6K in response to osmotic stress induced by sorbitol treatment of 293 cells. However these discrepancies may be a result of the use of different cell types or different means of inducing the cell stress.

The present work also shows that inhibition by mildly hypertonic conditions is not associated with acute dephosphorylation of p70S6K or Akt (Fig. 4). This is consistent with the reported dissociation of the kinetics of translation activity and p70S6K activity (as measured by S6 phosphorylation) in cells inhibited by or recovering from salt shock [38]. A recent report shows that mTOR remains active under moderately hypertonic conditions [33]. Thus it is unlikely that regulation of mTOR or its downstream targets is responsible for the effect of high salt on translation. Current evidence suggests a role for the protein kinases MEK1/2 in the regulation of protein synthesis by hypertonicity [41] but further work is clearly needed in this area.

As reported previously [20], [29], mTOR inhibitors have a relatively small effect on protein synthesis in cells under non-stressed conditions. Active site inhibitors such as Ku-0063794 and PP242 are more potent than rapamycin [29], [30], but even these compounds did not inhibit translation by more than 30% in exponentially growing MEFs (Fig. 1). Similar findings were obtained with the dual PI3-kinase/mTOR inhibitor PI-103 (Fig. 3). Such observations suggest that in exponentially growing cells the synthesis of the majority of proteins shows little requirement for free eIF4E (i.e. the form of the factor that can be sequestered by the 4E-BPs). Indeed it has been shown that 80% knockdown of eIF4E only inhibits global protein synthesis by 20% [43]. The basis for this remains to be established but one possibility is the functional “circularization” of polysomal mRNA [21], so that terminating ribosomes may be able to reattach to the 5′ end because the mRNA remains associated with eIF4G, with the need for cap recognition by eIF4E being by-passed altogether [44]. In contrast, under conditions where polypeptide chain initiation is disrupted by stress such reattachment may be inhibited, thus establishing a greater requirement for free eIF4E for reinitiation. Consistent with this, mRNA has been shown to dissociate from ribosomes and accumulate as free mRNP complexes under hypertonic conditions [45]. The increased dependency on eIF4E is not specific to salt treatment since we have shown that the stress of serum deprivation also sensitizes cells to the effects of mTOR inhibitors. However a number of other stress conditions, including DNA damage, endoplasmic reticulum stress and chemical hypoxia, do not appear to sensitize cells to Ku-0063794 (our unpublished data). There are several putative mechanisms for the lower probability of mRNA-ribosome binding under certain stress conditions, such as reduced stability of protein-protein interactions or protein-mRNA interactions, and these require further investigation to establish the basis for the stress-induced sensitivity to mTOR inhibitors.

The experiments reported here concern the control of overall protein synthesis and do not address the issue of likely variations in the translation of individual mRNA species in response to cell stress and/or mTOR inhibition. Earlier studies have shown that hypertonic conditions or the recovery from such conditions have differential effects on the synthesis of different proteins [46]. Likewise, mTOR inhibitors impair the translation of some mRNAs much more than that of others [47], with mRNAs possessing a 5′ terminal oligopyrimidine sequence or similar motif being particularly sensitive [30], [48]. Recently it has been shown that the translation of mRNAs encoding proteins involved in the invasive and metastatic properties of cancer cells is directly regulated by mTOR [49]. On the other hand, the mRNA encoding the p53 regulator mdm2 is particularly rapamycin-resistant [50]. The protocol of mild hypertonicity and mTOR inhibitor treatment described in this paper now provides a means to assess the relative eIF4E requirements of mRNAs with different 5′ UTRs, using either transfection of reporter constructs or a more global approach involving ribosome profiling and micro-array analysis [51].

Many previous studies have established the key role of the 4E-BPs as regulators of protein synthesis downstream of mTOR, and our demonstration that there is an absolute requirement for 4E-BP1/2 for the response to inhibitors of mTOR is in accord with this. It also shows that the likely presence (and apparent dephosphorylation) of 4E-BP3 is not sufficient to compensate for the lack of 4E-BP1 and 4E-BP2 in sensitizing cells to mTOR inhibition. In addition, our data show that the decreased phosphorylations of p70S6K and Akt that occur when mTORC1/2 activity is inhibited are not sufficient to affect overall protein synthesis, at least in the short term. Thus these proteins (as well as other mTOR substrates [52]) are unlikely to play a role in the acute control of overall translation. However we wish to emphasize that, whatever the relative importance of the various mTOR targets for the control of translation, none of these proteins – including 4E-BP1 and 4E-BP2– is effective under optimal growth conditions, at least in fibroblasts. Only under conditions of stress such as hypertonicity or serum deprivation does a role for the 4E-BPs become relevant. The lack of effect of p70S6K and Akt on overall translation is perhaps surprising since these protein families have several direct or indirect targets with potential roles in the regulation of protein synthesis [53]. Nevertheless a previous report [54] has also demonstrated that inhibition of p70S6K by rapamycin is not sufficient to inhibit cap-dependent translation. In the case of Akt, targets include not only mTOR itself [55] but also many other factors that control cell proliferation and survival (reviewed in [37]). The lack of a major effect of Akt on the protein synthetic machinery is suggested by the relatively small effect of PI-103 on translation under normal salt conditions, in spite of the fact that this compound has profound effects on Akt activity as a consequence of its inhibition of PI3-kinase [56]. However, our results do not rule out an important role for mTOR targets other than the 4E-BPs in the longer term effects of mTOR inhibitors on translation. Indeed, 24 h exposure to Ku-0063794 under normal salt conditions did cause a 30% reduction in protein synthesis in the DKO cells (versus 55% inhibition in 4E-BP wild-type cells) (data not shown). It is also possible that changes in the phosphorylation state of p70S6K and/or Akt and their targets may be important for the rapid regulation of the translation of individual mRNA species, via mechanisms that are independent of the 4E-BPs. In this connection it is of interest that Ku-0063794 did cause a partial impairment of eIF4F complex formation in the DKO cells (Fig. 5).

Recent reports using cell lines in which the levels of 4E-BP1 and 4E-BP2 have been experimentally manipulated show that these proteins play important roles in the regulation of cell proliferation [57], contact inhibition [58] and p53-dependent cell senescence [24]. In contrast, the regulation of cell growth (as opposed to proliferation) by mTOR does not involve the eIF4E binding proteins but does require S6 kinase activity [57]. It is probable that the control of cell proliferation by the 4E-BPs is a reflection of changes in the synthesis of key regulatory proteins whose mRNAs have a high requirement for eIF4E. Nevertheless our present data also indicate a more general role for the 4E-BPs in the control of overall protein synthesis under conditions of stress. These results may be of significance for our understanding of the role of the 4E-BPs in cancer. Physiological stress conditions often prevail in tumours *in vivo* due to lack of oxygen and nutrient supplies and malignant cells can evolve strategies to overcome such adverse conditions. In view of the importance of the 4E-BPs for the control of proliferation of untransformed cells, as well as the well known role of dysregulation of the eIF4E/4E-BP system in cancer progression [59], [60], it would be of interest to determine whether the sensitivity of protein synthesis and cell proliferation to mTOR inhibitors under stress conditions is diminished in transformed cells relative to their normal counterparts and whether this is determined by the relative levels of eIF4E versus the 4E-BPs in these cells. Thus the higher levels or activity of eIF4E often found in tumour cells may not only enhance the synthesis of growth-promoting or anti-apoptotic proteins (translated from relatively “weak” mRNAs) but also desensitize the cells to physiological stresses and the inhibition of mTOR. These considerations may provide guidelines for predicting the extent to which different kinds of tumour cells, particularly those which over-express eIF4E or have deregulated PI3K, Akt or mTOR activity, are likely to respond to the new generation of mTOR inhibitors and eIF4F disrupting agents that are now being developed for use in cancer therapy [44], [61]–[63].
